# Involvement of AMP-activated protein kinase in TGF-β-stimulated VEGF synthesis in osteoblasts

**DOI:** 10.3892/ijmm.2012.893

**Published:** 2012-01-23

**Authors:** JUN MIZUTANI, HARUHIKO TOKUDA, RIE MATSUSHIMA-NISHIWAKI, KENJI KATO, AKIRA KONDO, HIDEO NATSUME, OSAMU KOZAWA, TAKANOBU OTSUKA

**Affiliations:** 1 Department of Orthopedic Surgery, Nagoya City University Graduate School of Medical Sciences, Mizuho-Cho, Mizuho-Ku, Nagoya 467-8601; 2 Department of Pharmacology, Gifu University Graduate School of Medicine, Gifu 501-1194; 3 Department of Clinical Laboratory, National Center for Geriatrics and Gerontology, Morioka-Cho, Obu 474-8511, Japan

**Keywords:** adenosine 5′-monophosphate-activated protein kinase, transforming growth factor-β, mitogen-activated protein kinase, vascular endothelial growth factor, osteoblast

## Abstract

It is generally recognized that AMP-activated protein kinase (AMPK) acts as a key regulator of energy homeostasis. We have previously shown that transforming growth factor-β (TGF-β) stimulates synthesis of vascular endothelial growth factor (VEGF) via p44/p42 mitogen-activated protein (MAP) kinase, stress-activated protein kinase/c-*Jun* N-terminal kinase (SAPK/JNK) and p38 MAP kinase in osteoblast-like MC3T3-E1 cells. In the present study, we investigated whether AMPK is involved in the TGF-β-stimulated VEGF synthesis in osteoblast-like MC3T3-E1 cells. TGF-β time-dependently induced the phosphorylation of the AMPK α-subunit (Thr172) and the AMPK β-subunit (Ser108). Compound C, an AMPK inhibitor, significantly reduced the TGF-β-stimulated VEGF release. The inhibitory effect of compound C was also observed in normal human osteoblasts (NHOst). Although compound C failed to affect the TGF-β-induced phosphorylation of SAPK/JNK, p38 MAP kinase or Smad2, it markedly suppressed the TGF-β-induced phosphorylation of both MEK1/2 and p44/p42 MAP kinase. In addition, compound C significantly suppressed the VEGF mRNA expression induced by TGF-β. Taken together, our results strongly suggest that AMPK is involved in TGF-β-stimulated VEGF synthesis, and that it functions at a point upstream of MEK1/2.

## Introduction

Vascular endothelial growth factor (VEGF) is a potent mitogen displaying high specificity for vascular endothelial cells (1). VEGF, which is produced and secreted from a variety of cell types, increases capillary permeability and stimulates proliferation of endothelial cells (1). It is well known that the metabolism of bone tissue in the mammalian skeleton is a highly coordinated process of bone formation and bone resorption, regulated by osteoblasts and osteoclasts, respectively (2). The microvasculature is provided by capillary endothelial cells during bone remodeling. It is currently recognized that the activities of osteoblasts, osteoclasts, and capillary endothelial cells are closely coordinated, and properly regulate bone metabolism (3). These functional cells are considered to influence one another via humoral factors as well as by direct cell-to-cell contact. As for the regulation by VEGF of bone metabolism, it has been reported that an inactivation of VEGF causes complete suppression of blood vessel invasion concomitant with impaired trabecular bone formation and expansion of hypertrophic chondrocyte zone in mouse tibial epiphyseal growth plates (4). Osteoblasts synthesize VEGF in response to various physiological agents including transforming growth factor-β (TGF-β) (5). We have shown that the TGF-β-stimulated VEGF synthetic cascade is positively regulated via mitogen-activated protein (MAP) kinase superfamily such as p44/p42 MAP kinase, p38 MAP kinase and stress-activated protein kinase/c-*Jun* N-terminal kinase (SAPK/JNK) in osteoblast-like MC3T3-E1 cells (6,7). More recently, we have reported that Rho kinase negatively regulates the TGF-β-stimulated VEGF synthesis in MC3T3-E1 cells (8). These findings lead us to speculate that various kinds of intracellular molecules could modulate TGF-β-stimulated VEGF synthesis in osteoblasts. However, the exact mechanism underlying VEGF synthesis in osteoblasts has not yet been clarified.

Adenosine 5′-monophosphate-activated protein kinase (AMPK) is generally known to regulate multiple metabolic pathways (9). AMPK, defined as a mammalian protein kinase that was allosterically activated by AMP and was able to phosphorylate and inactivate enzymes of lipid synthesis (10), has emerged over the last decade as a key sensing mechanism in the regulation of cellular energy homeostasis (11–13). AMPK is activated in mammalian cells by a variety of physiological and pathological stresses that increase the intracellular AMP: ATP ratio, either by increasing ATP consumption or by decreasing ATP production. Compound C, a pyrrazolopyrimidine derivative which competitively inhibits AMPK, has been widely used as a specific and reversible AMPK inhibitor (14–16).

Activated AMPK acts to restore cellular energy balance by ATP generating pathways such as fatty acid oxidation, while simultaneously inhibiting ATP utilizing pathways. In addition to these functions as a metabolic regulator, it has been demonstrated that activated AMPK regulates bone formation and bone mass *in vitro* (17). However, the precise function of AMPK in the regulation of bone metabolism has not been fully elucidated.

In the present study, we investigated whether AMPK is involved in the TGF-β-stimulated VEGF synthesis in osteoblasts. We here show that AMPK is involved in TGF-β-stimulated VEGF synthesis in osteoblasts, and that AMPK acts at a point upstream of MEK1/2.

## Materials and methods

### Materials

Normal human osteoblasts (NHOst) were purchased from Cambrex (Charles, IA). TGF-β and mouse or human VEGF enzyme-linked immunosorbent assay (ELISA) kit were purchased from R&D Systems, Inc. (Minneapolis, MN). Compound C, a pyrrazolopyrimidine derivative which competitively inhibits AMPK (16), was purchased from Calbiochem, Inc. (San Diego, CA). Phospho-specific AMPKα (Thr172) and (Ser485) antibodies, AMPKα antibodies, phospho-specific AMPKβ (Ser108) and (Ser182) antibodies, AMPKβ, phospho-specific Smad2, Smad2, phospho-specific p44/p42 MAP kinase, p44/p42 MAP kinase, phospho-specific MEK1/2 and MEK1/2 antibodies were purchased from Cell Signaling Technology, Inc. (Beverly, MA). Glyceraldehyde-3-phosphate dehydrogenase (GAPDH) antibodies were obtained form Santa Cruz Biotechnology, Inc. (Santa Cruz, CA). The ECL western blotting detection system was purchased from GE Healthcare UK Ltd. (Buckinghamshire, UK). Other materials and chemicals were obtained from commercial sources.

### Cell culture

Cloned osteoblast-like MC3T3-E1 cells derived from newborn mouse calvaria (18) were maintained as previously described (19). Briefly, the cells were cultured in α-minimum essential medium (α-MEM) containing 10% fetal calf serum (FCS) at 37°C in a humidified atmosphere of 5% CO_2_/95% air. The cells were seeded into 35-mm diameter dishes (5×10^4^/dish) or 90-mm diameter dishes (20×10^4^/dish) in α-MEM containing 10% FCS. After 5 days, the medium was exchanged to α-MEM containing 0.3% FCS and incubated for 48 h. The cells were then used for subsequent experiments.

NHOst cells were seeded into 35-mm (5×10^4^/dish) diameter dishes in α-MEM containing 10% FCS (20). After 6 days, the medium was changed to α-MEM containing 0.3% FCS. The cells were used for experiments after 48 h.

### VEGF assay

The cultured cells were pretreated with various doses of compound C for 60 min, and then stimulated by 5 ng/ml TGF-β or vehicle in 1 ml of α-MEM containing 0.3% FCS for 48 h. The conditioned medium was collected at the end of the incubation, and the VEGF concentration was measured by cell species-responsible ELISA kits. The absorbance of enzyme-linked immunosorbent assay (ELISA) samples was measured at 450 nm with the EL 340 Bio Kinetic Reader (Bio-Tek Instruments, Inc., Winooski, VT) according to the manufacturer’s protocol.

### Real-time RT-PCR

The cultured cells were pretreated with 10 μM compound C or vehicle for 60 min, and then stimulated by 5 ng/ml TGF-β in a-MEM containing 0.3% FCS for the indicated periods. Total-RNA was isolated and transcribed into complementary DNA using TRIzol reagent (Invitrogen Corp., Carlsbad, CA) and Omniscript reverse transcriptase kit (Qiagen, Inc., Valencia, CA), respectively. Real-time RT-PCR was performed using a Light Cycler system in capillaries and FastStart DNA Master SYBR-Green I provided with the kit (Roche Diagnostics, Basel, Switzerland). Sense and antisense primers were synthesized based on the report of Simpson *et al* (21) for mouse VEGF mRNA. Sense and antisense primers for mouse VEGF mRNA were purchased from Takara Bio., Inc. (Tokyo, Japan) (primer set ID: MA039013). The amplified products were determined by melting curve analysis and agarose electrophoresis. VEGF mRNA levels were normalized with those of GAPDH mRNA.

### Western blot analysis

Western blot analysis was performed as previously described (22). In brief, the cultured cells were pretreated with various doses of compound C for 60 min, and then stimulated by 5 ng/ml TGF-β or vehicle in α-MEM containing 0.3% FCS for the indicated periods. The cells were washed twice with phosphate-buffered saline and then lysed, homogenized and sonicated in a lysis buffer containing 62.5 mM Tris/HCl, pH 6.8, 3% sodium dodecyl sulfate (SDS), 50 mM dithiothreitol and 10% glycerol. SDS-polyacrylamide gel electrophoresis (PAGE) was performed according to Laemmli (23) in 10% polyacrylamide gels. The protein (10 μg) was fractionated and transferred onto an Immuno-Blot PVDF membrane (Bio-Rad, Hercules, CA). Membranes were blocked with 5% fat-free dry milk in Tris-buffered saline-Tween-20 (TBS-T; 20 mM Tris/HCl, pH 7.6, 137 mM NaCl, 0.1% Tween-20) for 2 h before incubation with the primary antibodies. Peroxidase-labeled antibodies raised in goat against rabbit IgG were used as second antibodies. The primary and secondary antibodies were diluted at 1:1,000 with 5% fat-free dry milk in TBS-T. Peroxidase activity on the membrane was visualized on X-ray film by means of the ECL western blotting detection system.

### Statistical analysis

The data were analyzed by ANOVA followed by the Bonferroni method for multiple comparisons between pairs, and a P<0.05 was considered statistically significant. All data are presented as the mean ± SEM of triplicate independent determinations.

## Results

### Effects of TGF-β on the phosphorylation of AMPK subunits in MC3T3-E1 cells

**Figure 1:**
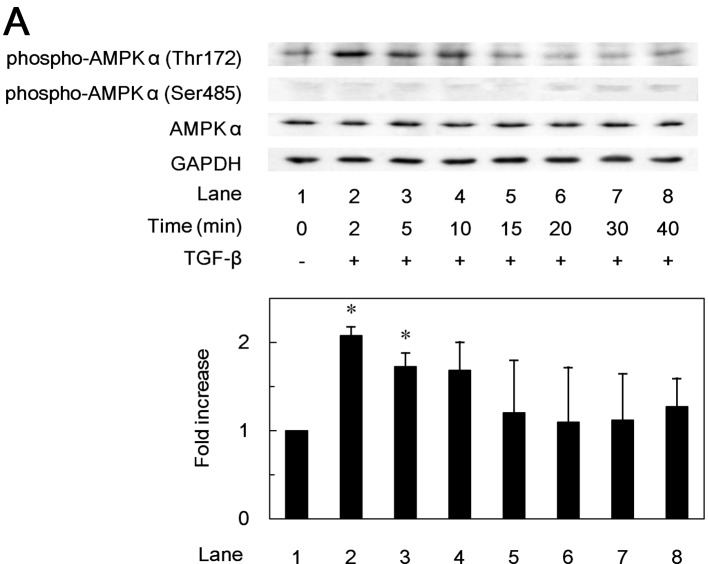

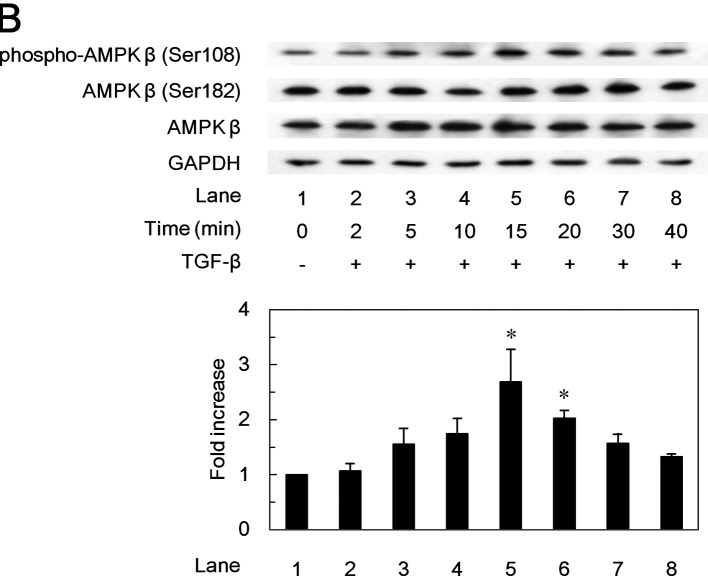
Effects of TGF-β on the phosphorylation of (A) AMPK α-subunits and (B) AMPK β-subunits in MC3T3-E1 cells. The cultured cells were stimulated by 5 ng/ml TGF-β for the indicated periods. The extracts of cells were subjected to SDS-PAGE with subsequent western blot analysis with antibodies against phospho-specific AMPKα (Thr172), phospho-specific AMPKα (Ser485) antibodies, AMPKα antibodies, phospho-specific AMPKβ (Ser108), phospho-specific AMPKβ (Ser182), AMPKβ or GAPDH antibodies. The histgram shows quantitative representations of the levels of TGF-β induced phosphorylation obtained from laser densitometric analysis of three independent experiments. Each value represents the mean ± SEM of triplicate determinations. ^*^P<0.05, compared to the value of lane 1.

In order to investigate whether TGF-β stimulates the activation of AMPK in osteoblast-like MC3T3-E1 cells, we first examined the effects of TGF-β on the phosphorylation of the AMPK subunits. TGF-β markedly induced the phosphorylation of AMPK α-subunit (Thr172) in a time-dependent manner (Fig. 1A). The phosphorylation of the AMPK α-subunit (Thr172) by TGF-β reached its maximum at 2 min, and decreased thereafter. TGF-β also time-dependently induced the phosphorylation of the AMPK β-subunit (Ser108) (Fig. 1B). The phosphorylation level of AMPK β-subunit (Ser108) reached a peak at 15 min, and decreased thereafter. However, TGF-β had little effect on the phosphorylation of AMPK α-subunit (Ser485) or AMPK β-subunit (Ser182) (Fig. 1).

### Effect of compound C on the TGF-β-stimulated VEGF synthesis in MC3T3-E1 cells

**Figure 2:**
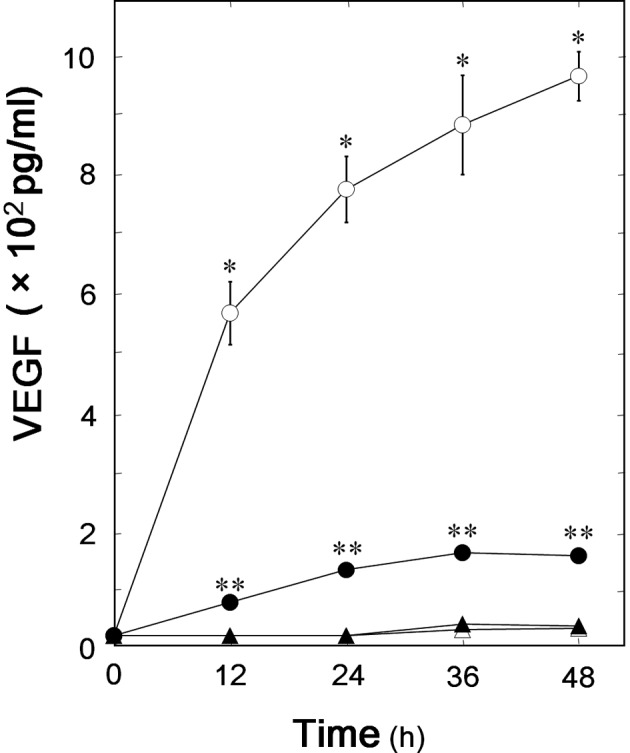
Effect of the AMPK inhibitor on the TGF-β-stimulated VEGF synthesis in MC3T3-E1 cells. The cultured cells were pretreated with 10 μM AMPK inhibitor (●, ▴) or vehicle (○, ▵) for 60 min, and then stimulated by 5 ng/ml TGF-β (circles) or vehicle (triangles) for the indicated periods. Each value represents the mean ± SEM of three experiments performed by different cell preparations. ^*^P<0.05, compared to the value of control; ^**^P<0.05, compared to the value of TGF-β alone.

**Figure 3:**
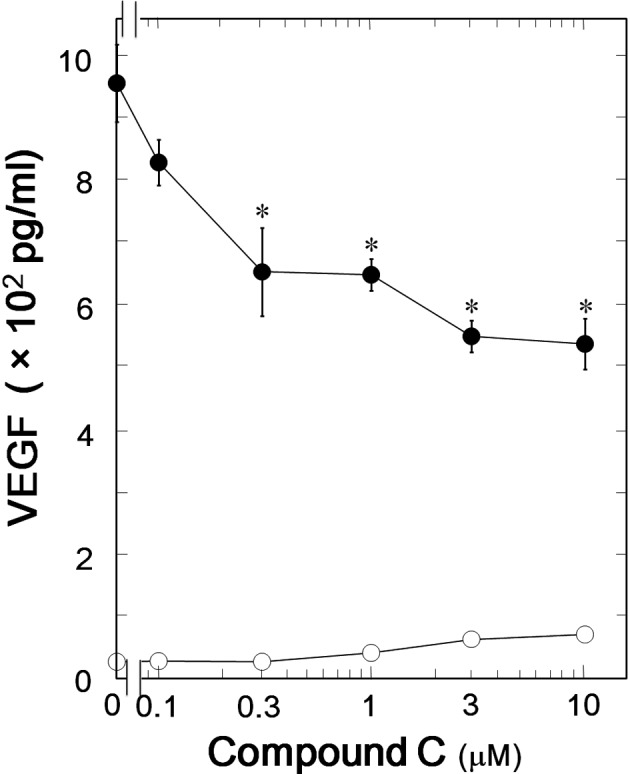
Effect of the AMPK inhibitor on the TGF-β-stimulated VEGF synthesis in MC3T3-E1 cells. The cultured cells were pretreated with various doses of AMPK inhibitor for 60 min, and then stimulated by 5 ng/ml TGF-β (●) or vehicle (○) for 48 h. Each value represents the mean ± SEM of three experiments performed by different cell preparations. ^*^P<0.05, compared to the value of the control.

We have previously showed that TGF-β significantly stimulates VEGF synthesis in osteoblast-like MC3T3-E1 cells (6). To investigate whether AMPK is involved in the TGF-β-stimulated VEGF synthesis in MC3T3-E1 cells, we next examined the effect of compound C, an inhibitor of AMPK, on VEGF synthesis stimulated by TGF-β. Compound C significantly suppressed the TGF-β-induced VEGF synthesis (Fig. 2). The inhibitory effect of compound C on the VEGF synthesis was dose-dependent in the range between 1 and 10 μM (Fig. 3). The maximum effect of compound C was observed at 10 μM and caused approximately 40% suppression in the TGF-β-effect.

### Effect of compound C on the TGF-β-stimulated VEGF synthesis in NHOst cells

**Figure 4:**
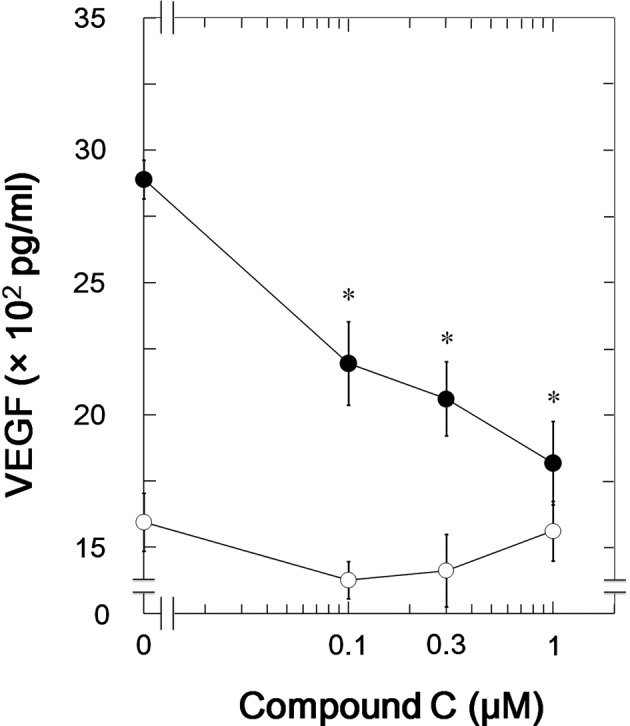
Effect of the AMPK inhibitor (compound C) on the TGF-β-stimulated VEGF synthesis in NHOst cells. The cultured cells were pretreated with various doses of AMPK inhibitor for 60 min, and then stimulated by 5 ng/ml TGF-β (●) or vehicle (○) for 48 h. Each value represents the mean ± SEM of three experiments performed by different cell preparations. ^*^P<0.05, compared to the value of TGF-β alone.

We next examined the effects of compound C in NHOst, a different osteoblastic cell line. Compound C, as well as in MC3T3-E1 cells, significantly suppressed the TGF-β-induced VEGF synthesis in NHOst (Fig. 4). The inhibitory effect of compound C on the VEGF synthesis was dose-dependent in the range between 0.1 and 1 μM. The maximum effect of compound C was observed at 1 μM and caused approximately 80% suppression in the TGF-β-effect.

### Effect of compound C on the TGF-β-induced expression levels of VEGF mRNA in MC3T3-E1 cells

**Figure 5:**
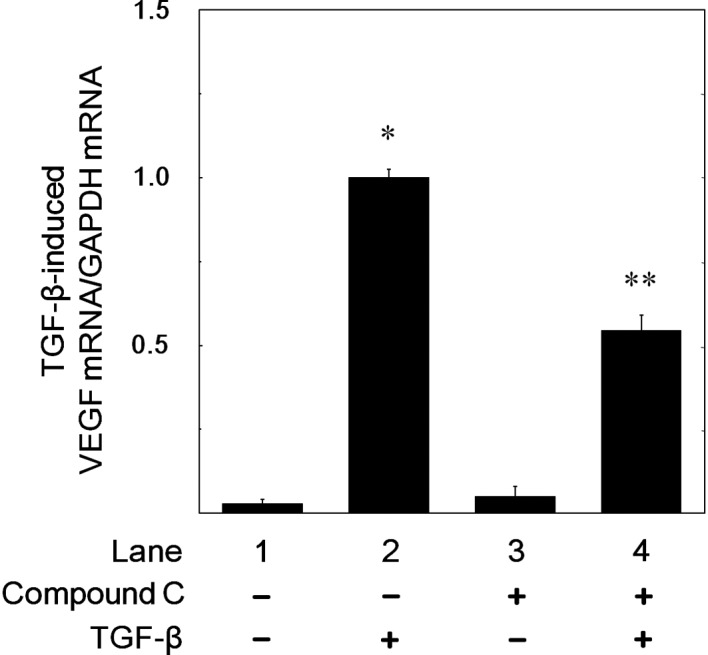
Effect of AMPK inhibitor on the TGF-β-stimulated expression levels of VEGF mRNA in MC3T3-E1 cells. The cultured cells were pretreated with 10 *μ*M AMPK inhibitor or vehicle for 60 min, and then stimulated by 5 ng/ml TGF-β for 12 h. The respective total-RNA were then isolated and quantified by real-time RT-PCR. Results were standardized for the value of control. Each value represents the mean ± SEM of three independent determinations performed by a single cell preparation. ^*^P<0.05, compared to the value of control; ^**^P<0.05, compared to the value of TGF-β alone.

It has previously been reported that TGF-β induces VEGF mRNA expression in MC3T3-E1 cells (5). In order to clarify whether AMPK affects TGF-β-stimulated VEGF release through the modulation of a transcriptional event or not, we furthermore examined the effect of compound C on the TGF-β-induced VEGF mRNA expression. We confirmed that VEGF mRNA expression levels induced by TGF-β were increased in a time-dependent manner in accordance with a previous report (5). Compound C (10 μM) significantly suppressed the TGF-β-induced VEGF mRNA expression levels (Fig. 5). Compound C of 10 μM caused approximately 50% inhibition in the TGF-β-effect. We confirmed that GAPDH mRNA was constitutively expressed and stable in MC3T3-E1 cells (data not shown).

### Effects of compound C on the phosphorylation of Smad2 or p44/p42 MAP kinase induced by TGF-β in MC3T3-E1 cells

**Figure 6:**
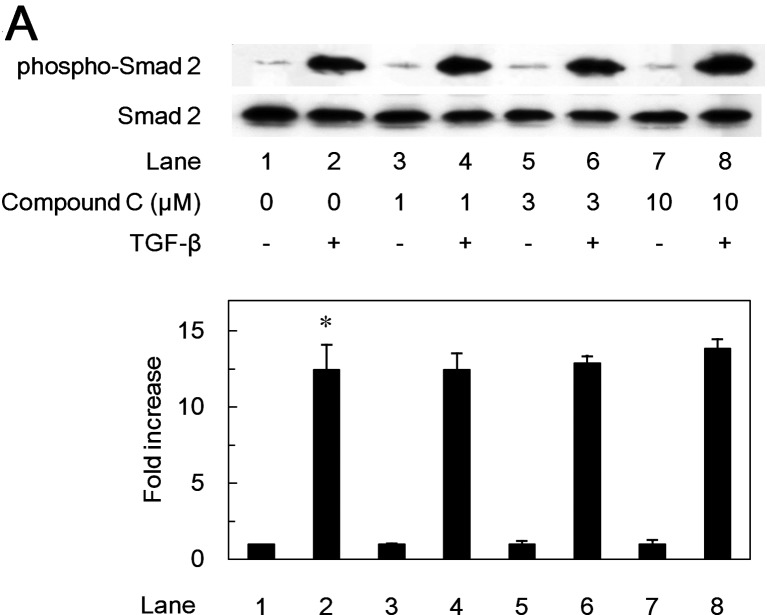

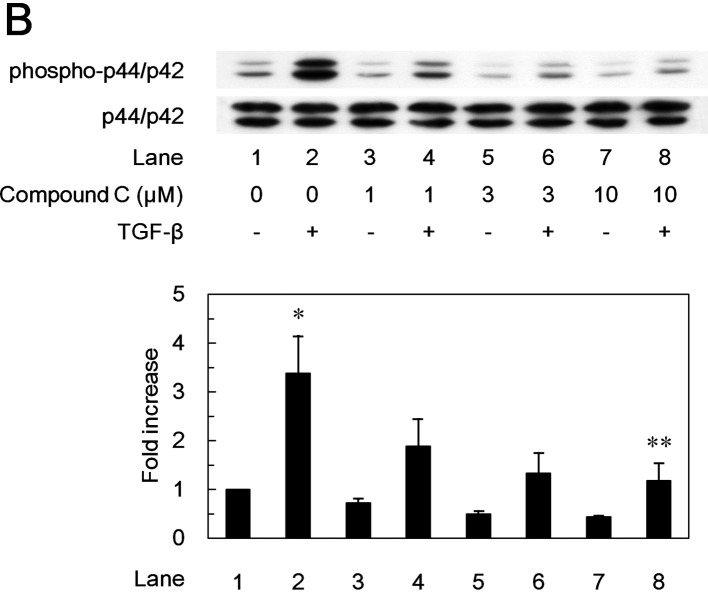

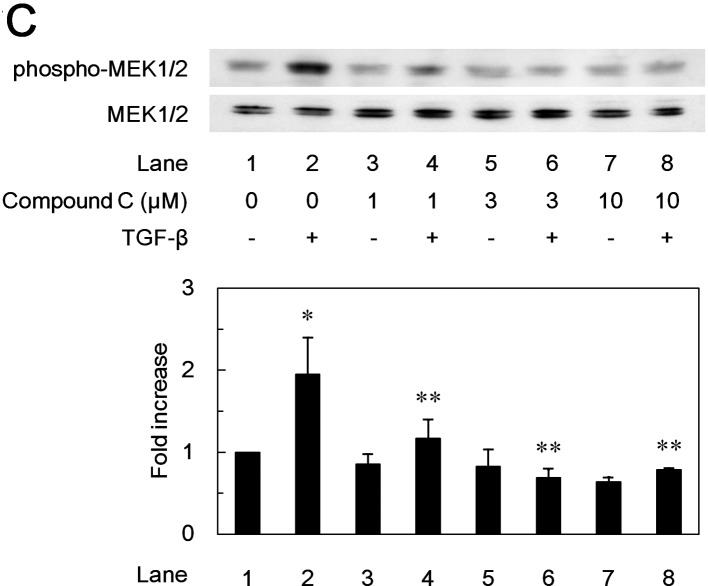
Effects of AMPK inhibitor on the TGF-β-induced phosphorylation of (A) Smad2, (B) p44/p42 MAP kinase and (C) MEK1/2 in MC3T3-E1 cells. The cultured cells were pretreated with various doses of AMPK inhibitor for 60 min, and then stimulated with 5 ng/ml TGF-β or vehicle for 120 min. The cell extracts were then subjected to SDS-PAGE with subsequent western blot analysis with antibodies against phospho-specific Smad2, Smad2, phospho-specific p44/p42 MAP kinase, p44/p42 MAP kinase, phospho-specific MEK1/2 and MEK1/2. The histogram shows quantitative representations of the levels of TGF-β induced phosphorylation obtained from laser densitometric analysis of three independent experiments. Each value represents the mean ± SEM of triplicate determinations. ^*^P<0.05, compared to the value of lane 1; ^**^P<0.05, compared to the value of lane 2.

It is well known that receptor-activated Smads such as Smad2 are principal mediators of intracellular signaling from TGF-β receptors to the nucleus (24). Thus, we next examined the effect of compound C on the TGF-β-induced phosphorylation of Smad2. However, compound C failed to affect the TGF-β-induced phosphorylation level of Smad2 in the range between 1 and 10 μM (Fig. 6A).

It is currently recognized that other proteins mediate the intracellular signaling by TGF-β in addition to Smads (25). We have previously demonstrated that p44/p42 MAP kinase, p38 MAP kinase and SAPK/JNK are involved in the TGF-β-stimulated VEGF synthesis in osteoblast-like MC3T3-E1 cells (6,7). In addition, we examined the effects of compound C on the TGF-β-induced phosphorylation of p44/p42 MAP kinase, p38 MAP kinase or SAPK/JNK. Compound C markedly suppressed the TGF-β-induced phosphorylation level of p44/p42 MAP kinase in a dose-dependent manner between 1 and 10 μM (Fig. 6B). Furthermore, compound C also suppressed TGF-β-stimulated phosphorylation of MEK1/2, an upstream kinase that activates p44/p42 MAPK (Fig. 6C). On the other hand, compound C had little effect on the phosphorylation of p38 MAP kinase or SAPK/JNK induced by TGF-β (data not shown).

## Discussion

AMPK exists as heterotrimeric complex comprising a catalytic α-subunit and regulatory β- and γ-subunits (9). Phosphorylation of the Thr172 residue in the α-subunit is essential for AMPK activation (26). Once activated, AMPK causes an over 100-fold increase in kinase activity (27,28). A carbohydrate binding module (CBM) is located within the central region of the β-subunit (9). In the CBM, multiple autophosphorylation sites including Ser108 have been identified (29). In the present study, we demonstrated that the maximum phosphorylation of the AMPK α-subunit (Thr172) occurred at 5 min, followed by a peak phosphorylation of the AMPK β-subunit (Ser108) at 15 min. Although further examination is warranted to elucidate the precise mechanism of the relationship between AMPK activation and TGF-β stimulation, our results strongly suggest that TGF-β triggers phosphorylation of AMPK followed by a series of activation events of AMPK for TGF-β-stimulated VEGF synthesis in osteoblasts. In addition, we found that compound C (15,16) also suppressed TGF-β-induced VEGF synthesis in NHOst cells. It is likely that AMPK is involved in TGF-β-induced VEGF synthesis also on human osteoblasts. To the best of our knowledge, this is the first report showing the involvement of AMPK in TGF-β-stimulated VEGF synthesis in osteoblasts.

It is well known that TGF-β mainly employs receptor-activated Smad proteins such as Smad2 and Smad3 as intracellular mediators of signaling (30). However, compound C, an AMPK inhibitor, had no effect on the TGF-β-induced phosphorylation of Smad2 in osteoblast-like MC3T3-E1 cells. Therefore, it seems unlikely that AMPK acts at a point upstream of Smad2 in the VEGF synthesis in MC3T3-E1 cells. It is currently recognized that TGF-β exerts its effects on a variety of biological functions via Smad-independent signaling in addition to Smad-dependent signaling (25). The MAP kinase superfamily, such as the p44/p42 MAP kinase, p38 MAP kinase and SAPK/JNK act as central elements used by mammalian cells to transduce the various extracellular messages (24,31). As for TGF-β-stimulated VEGF synthesis in osteoblast-like MC3T3-E1 cells, we have reported the involvement of p44/p42 MAP kinase, p38 MAP kinase and SAPK/JNK (6,7). Thus, we investigated the effects of compound C on the TGF-β-induced phosphorylation of these three MAP kinases. Compound C had little effect on the phosphorylation of SAPK/JNK or p38 MAP kinase induced by TGF-β. In contrast, TGF-β-induced phosphorylation of both p44/42 MAP kinase and MEK1/2 was markedly suppressed by compound C. These findings strongly suggest that AMPK acts at a point upstream of MEK1/2 in the TGF-β-stimulated VEGF synthesis in osteoblast-like MC3T3-E1 cells. TGF-β-activated kinase (TAK1), a member of the MAP kinase kinase kinase family, has been identified as an upstream kinase of MAP kinase including osteoblast-like MC3T3-E1 cells (32). It has been recently reported that expression of TAK1 in HeLa cells stimulates phosphorylation of the AMPK α-subunit (Thr172) (33). Based on these findings, it is most likely that AMPK may act as a regulator at the point between TAK1 and MEK1/2 in TGF-β-stimulated VEGF synthesis in osteoblast-like MC3T3-E1 cells. Recent reports have indicated that the possibility of AMPK-independent effects exerted by compound C (16,34,35). Thus, it is possible that an AMPK-independent mechanism is involved in compound C-induced suppression of TGF-β-stimulated VEGF synthesis in osteoblasts. However, there is no available inhibitor for AMPK which is more specific than compound C to the best of our knowledge. Further investigations would be required to clarify the details.

Recent studies have reported that both AMPK and VEGF play a significant role in bone metabolism (1,17,36,37). Germline deletion of either AMPKβ1 or β2 isoforms reportedly resulted in reduced trabecular bone density and mass without effects on osteoclast or osteoblast numbers, showing that AMPK is required to maintain normal bone density (38). Therefore, it is possible that TGF-β-induced VEGF synthesis via MEK1/2-p44/p42 MAP kinase regulated by AMPK plays an important role in the homeostasis of bone density under physiological conditions. Many hormones and neuromediators, including leptin, ghrelin, cannabinoids, and the sympathetic nervous system that regulate food intake and energy expenditure through AMPK activation also regulate bone mass (39–47). VEGF is critical for bone angiogenesis, and VEGF secreted from osteoblasts may play a pivotal role in the regulation of bone metabolism. It has been reported that TGF-β induces differentiation or proliferation of osteoblasts, and inhibits the formation of osteoclast precursors (48). Therefore, it is highly speculated that TGF-β-stimulated VEGF synthesis via AMPK acts as a positive regulator of bone remodeling and AMPK is probably a key molecule in bone metabolism as seen in fat metabolism. We have recently reported that AMPK positively regulates FGF-2-stimulated VEGF synthesis via both p44/p42 MAP kinase and SAPK/JNK in osteoblast-like MC3T3-E1 cells (49). It is likely that the regulatory mechanisms of VEGF synthesis by AMPK in osteoblasts are different in each stimulus, suggesting that the sophisticated regulation by AMPK is essential to promote the cellular event, VEGF synthesis. However, the exact role of AMPK in osteoblasts is not fully clarified. Further investigation is necessary to elucidate the role of AMPK in bone metabolism.

In conclusion, our results strongly suggest that AMPK functions at a point upstream of MEK1/2 and positively regulates TGF-β-stimulated VEGF synthesis via p44/p42 MAP kinase activation in osteoblasts.
